# Comparison of the Effects of Hand Reflexology versus Acupressure on Anxiety and Vital Signs in Female Patients with Coronary Artery Diseases

**DOI:** 10.3390/healthcare7010026

**Published:** 2019-02-11

**Authors:** Zohre Rahmani Vasokolaei, Nahid Rejeh, Majideh Heravi-Karimooi, Seyed Davood Tadrisi, Kiarash Saatchi, Zahra Poshtchaman, Christina Sieloff, Mojtaba Vaismoradi

**Affiliations:** 1 Department of Nursing, Faculty of Nursing and Midwifery, Shahed University, Tehran 3319118651, Iran; rahmanizohreh114@gmail.com; 2 Elderly Care Research Center, Department of Nursing, Faculty of Nursing and Midwifery, Shahed University, Tehran 3319118651, Iran; majidehheravi@yahoo.com; 3 Faculty of Nursing, Baqiyatallah University of Medical Sciences, Tehran 1435915371, Iran; sdt1344@gmail.com; 4 Iranian Scientific Acupuncture Association, Tehran 1414734117, Iran; drsaatchiclinic@gmail.com; 5 Department of Nursing, Sabzevar University of Medical Sciences, Sabzevar 9617913114, Iran; zahraposhtchaman@yahoo.com; 6 College of Nursing, Montana State University, Bozeman, MT 59715, USA; sieloffc@hotmail.com; 7 Faculty of Nursing and Health Sciences, Nord University, 8049 Bodø, Norway; mojtaba.vaismoradi@nord.no

**Keywords:** acupressure, complementary therapies, anxiety, coronary artery disease, hand reflexology, vital sign

## Abstract

Hospitalization in the cardiac care unit can increase anxiety in patients. This study aimed to compare hand reflexology versus acupressure on anxiety and vital signs in female patients with coronary artery diseases. This double-blinded randomized placebo-controlled trial with a pre- and post-intervention design was conducted on 135 female patients with coronary artery diseases. Female patients hospitalized in a cardiac care unit were randomly divided into three groups of hand reflexology, acupressure and placebo (*n* = 45 patients in each group) using blocking and a table of random numbers. Data was collected using the Spielberger anxiety inventory. Also, their vital signs were measured before, immediately after and half an hour after the intervention. Data analysis was performed using descriptive and analytical statistics. Before the intervention, there was no statistically significant difference in anxiety levels between the groups (*p* > 0.05). Also, the effects of hand reflexology and acupressure immediately and half an hour later on the reduction of anxiety and vital signs were equal (*p* < 0.05). Implementation of hand reflexology and acupressure can have positive effects on anxiety and vital signs in patients with coronary artery diseases. They can reduce patients’ anxiety with an equal effectiveness.

## 1. Introduction

Coronary artery disease (CAD) is the most common chronic and life threating disease [1] and is the most important cause of mortality and disability [2]. In the United States, around 1.5 million people annually suffer from myocardial infarction. Also, a large number of them are hospitalized resulting in significant economic losses due to frailty of such patients [3]. In developing countries, prevalence of CAD and related mortality are increasing [4].

Most patients with CAD are hospitalized in the cardiac care unit (CCU), and often suffer from high levels of anxiety due to being connected to the monitor, requesting catheter and bedpan, and environmental voices [5,6]. Furthermore, anxiety can predict harmful and dangerous cardiovascular outcomes in patients with CAD such as fatal and nonfatal cardiac events, hospital readmissions and cardiac mortalities [7]. Drugs such as benzodiazepines and tricyclic antidepressants, and nonpharmacological methods including motivational interviewing, are used to reduce anxiety in patients [8,9]. Medications have several complications and impose large amounts of costs on healthcare systems [10]. The overall cost of anxiety disorders has been estimated to be $1657.52 per person or $33.71 billion in total annually [11]. The safe management of patient anxiety is a major challenge and often is an obstacle to achieve desired patient outcomes [12]. There is a need for safe interventions for relieving and controlling patients’ anxiety. For instance, the use of nonpharmacological interventions such as complementary therapies has increased. Acupressure as a traditional complementary therapy is rooted in Chinese traditional medicine [13].

According to the National Medical Library of the United States, acupressure primarily focuses on the body’s specific areas with the aim of improving patients’ health [13]. While points used in acupressure are also used in acupuncture, no needles or similar things are used [14]. This traditional method acts by stimulating the secretion of neurotransmitters and adrenocorticotropin hormones using endorphin-mediated mechanisms [15]. Acupressure can significantly improve mental and psychological symptoms such as anxiety. Regarding the reduction of anxiety, studies have been conducted on patients undergoing ventilation support [16], having respiratory problems [17], before and after abdominal surgery and amputation [18,19]. Similar studies have been conducted on high school and college students [20,21], which have shown different and contradictory results.

One of the acupressure points used to relieve anxiety is point P6, which is located on the inner side of the forearm and in the hole between forearm bones and three finger widths above the wrist fold [22]. Application of massage at the point P6 alone can provide relaxation of the autonomic nerves of the heart. However, in combination with the Yintang point massage called the third eye, it can have a greater effect on mental relaxation [23]. 

Hand reflexology is a type of massage with a long history [24]. It has become more common in recent years and is recognized as a scientific process [25]. Hand reflexology of stress points not only is a simple massage, but also is a noninvasive and safe complementary therapy that involves a direct pressure on specific body points connecting specific organs. Subsequently, it can create an anesthetic effect on other body areas [26,27]. Hand reflexology can affect mental/emotional/psychological mechanisms. For instance, touching and contacting skin can release androgen endorphins in the body, which makes a person to feel both relaxed and re-energized [28,29]. Following the application of pressure, stress and anxiety messages are blocked within the body and activity of the sympathetic nervous system is diminished [30].

Studies on massage and reduction of anxiety in patients hospitalized in the CCU, and the associated effects of relaxation on individual physiological indices, have shown positive results [31]. Mahmoudirad et al. (2014) reported reductions in anxiety scores of patients receiving either foot reflexology or acupressure [32]. Bahrami et al. (2015) and Stephenson et al. (2007) identified the effectiveness of massage on the reduction of patients’ anxiety compared to the pre-intervention condition. However, no comparative examination on the effects of hand reflexology and acupressure on anxiety in patients with CAD has been performed [5,33]. Therefore, this study aimed to compare the effects of hand reflexology and acupressure on anxiety and vital signs in female patients hospitalized in the CCU. 

## 2. Methods

### 2.1. Design and Sample

A double-blind randomized placebo-controlled trial with a pre- and post-intervention design was used. A convenience sample of 135 female patients with CAD was randomly allocated into three groups of hand reflexology, acupressure in hands and placebo. Only female patients were recruited to prevent the effect of gender on the study outcome. Each patient was hospitalized for more than two days in the CCU and was diagnosed with CAD. The intervention groups received either hand reflexology or acupressure. The placebo group only received simple touch on thumbs without hand reflexology or acupressure stimulation. 

### 2.2. Ethical Considerations

The institutional review board approval was achieved from the university in which the second researcher worked. The research protocol was registered at the Iranian Registry of Clinical Trial website under the code of IRCT201703287529N13. The research purpose and method were described to the eligible patients. They were requested to sign the informed consent form. Also, numbers rather than names were used to de-identify the participants to ensure confidentiality and anonymity. While no harm was anticipated for the patients due to the complementary identity of the interventions, a cardiologist in the CCU cooperated to conduct them. No patients withdrew from the study and no harm was identified throughout the research process. 

### 2.3. Eligibility Criteria

The following inclusion criteria were used for recruitment of the patients: female patients diagnosed with CAD, age 18 years and older, no routine use of anxiolytic drugs, not being pregnant, no presence of infection, ulcers or skin diseases in hands, no history of drug addiction, having no cardiac pacemaker, no sensory impairments and no history of reflexology massage. Only female patients were recruited to eliminate any gender influence on the anxiety level. Exclusion criteria were the use of warfarin, refusal to complete the intervention session, being transferred to another unit, and contraindication to continue the intervention. 

### 2.4. Setting and Participants

This study was conducted in a high-turnover CCU of a university hospital in an urban area of Iran from March 2016 to July 2017. One hundred thirty women were chosen using a convenience sampling method. None of them were excluded from the study. They were randomized into one of three groups using blocking and a table of random numbers as follow: (i) acupressure (*n* = 45); (ii) hand reflexology (*n* = 45), and (iii) placebo (*n* = 45) groups.

### 2.5. Sample Size

Given the sample size in a previous study [34], α = 0.05, and power = 80%, 35 patients were needed to be recruited based on the following sampling formula:

(1) $$N\;=\frac{{\left(z/1-\frac{\alpha }{2}+z/\beta \right)}^{2}*\left(\sigma 1^{2}+\sigma 2^{2}\right)}{{\left(\mu 1-\mu 2\right)}^{2}}=\frac{{\left(1.96+0.85\right)}^{2}*\left(0.77^{2}+0.71^{2}\right)}{{\left(2.40-1.90\right)}^{2}}=34.65\;\approx \;35.$$

Also, given 30% probability of samples’ attrition (*n* = 10 in each group), the sample size was determined 135 persons (*n* = 45 in each group).

### 2.6. Random Allocation

After obtaining permission to enter the CCU, the nurse manager was informed of the purpose and inclusion criteria to help with the identification of eligible patients. A convenience sample of female patients was chosen with no patient declining to participate.

**Figure 1:**
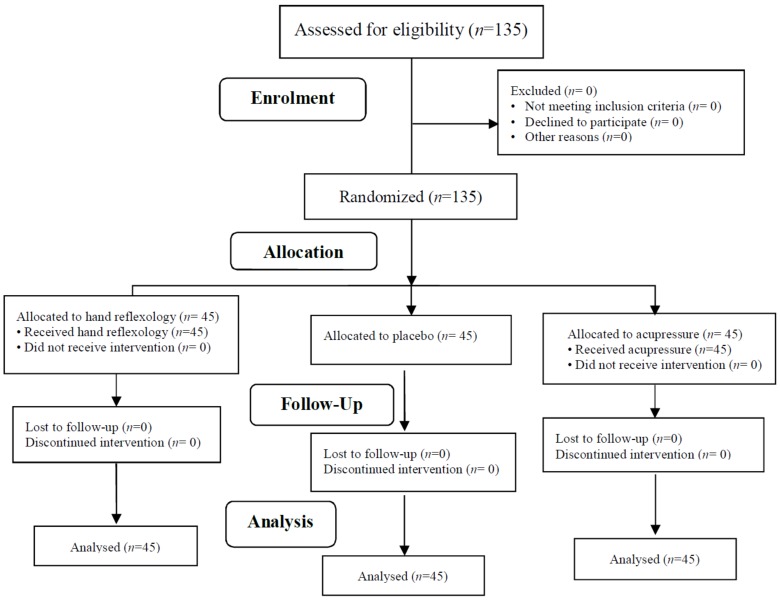
Process of the study according to the Consort flow diagram (2010).

Assignments to each group were generated using blocking and a table of random numbers. The size of blocks was not announced to prevent selection bias. In addition, blockings were available only to a research fellow who was not informed of the study process. To further avoid bias, the subjects and staff nurses responsible for data collection were blind to the random allocation process. The sampling process continued until a sufficient number of patients were recruited (Figure 1).

The participants’ codes were disclosed to the researchers after finalizing statistical data analysis. In addition, the data analyst was unaware of group assignments. 

### 2.7. Measurements

Data collection tools were the personal and medical data forms and the Spielberger’s State Trait Anxiety Inventory (STAI) [35]. 

The personal and medical data form contained questions about the patients’ age, length of hospitalization, education level, employment status, marital status, smoking, and history of hospitalization. It was completed using each patient’s medical records or also interviewing them.

Spielberger in 1970 developed the STAI as a self-administered questionnaire [35]. It consisted of 40 items divided into two subscales, which were completed through interviewing. The State Anxiety (SA) subscale (1–20) and the Trait Anxiety subscale had 20 items (21–40). The SA subscale asked a patient to describe their feelings in certain conditions. The TA scale asked them to describe how routinely they felt in typical situations that everyone experienced on a daily basis. The item scoring was from one to four. Positive items scored from very low (4), low (3), high (2), to very high (1). Negative items had a reverse scoring from very low (1), low (2), high (3), to very high (4). The total score was between 40 (the lowest possible anxiety) and 120 (the highest possible anxiety). A score of 40–79 indicated mild anxiety, 80–119 indicated moderate anxiety and 120–160 showed severe anxiety [36]. Validity and reliability of the Farsi version of the STAI were confirmed by Mahram (1994) [37]. Also, its validity and reliability was confirmed by Tiedeman and Clatworthy (1990). Reliability using the calculation of a Cronbach alpha coefficient was 0.82 [38].

Monitors were used to measure respiratory rate (RR), heart rate (HR), blood pressure (BP), and mean arterial pressure (MAP). Also, oxygen saturation (SPo2) was measured using a pulse oximetry device.

### 2.8. Interventions

Acupressure and hand reflexology interventions were carried out once and by the female researcher. She protected the participant’s privacy through curtains and asked visitors to leave the CCU during the interventions. She sat beside the patient on a chair and applied pressure so that they felt warm, heavy, swollen or numb at pressure points without any discomfort. 

### 2.9. Acupressure Group

**Figure 2:**
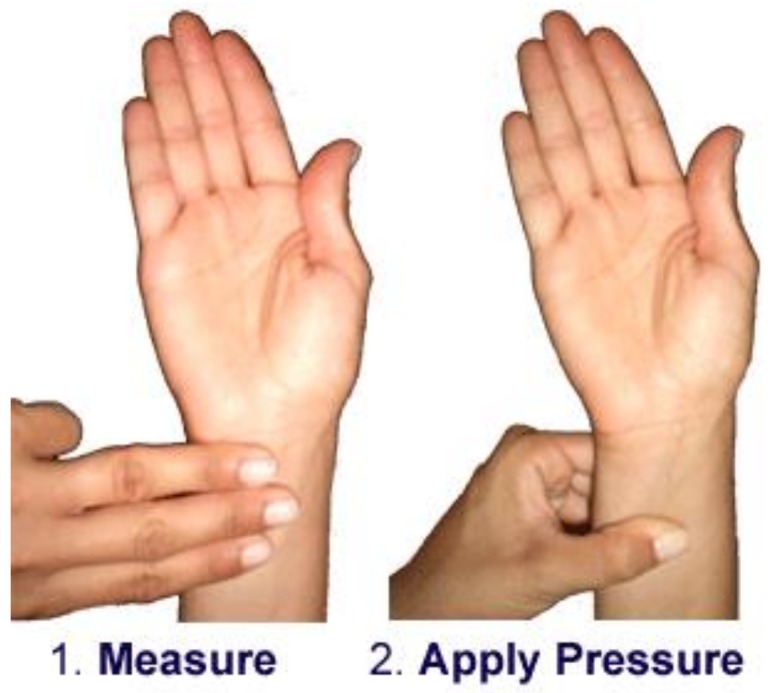
The point of pressure in the acupressure group.

Acupressure was performed for 10 min per hand on the right hand and then the left hand. Pressure was applied to the Nei Guan point (P6 or Inner Gate) on the inner side (ventral) of the forearm. The first pressure was applied two-and-one-half finger widths from the wrist crease. The pressure was applied approximately 2 inches lower toward the elbow (the points lay between the two tendons). Point P6 could then be stimulated on both hands through applying a firm pressure to the point using the index finger for one minute, while breathing shallow through the nose (Figure 2).

### 2.10. Hand Reflexology Group

**Figure 3:**
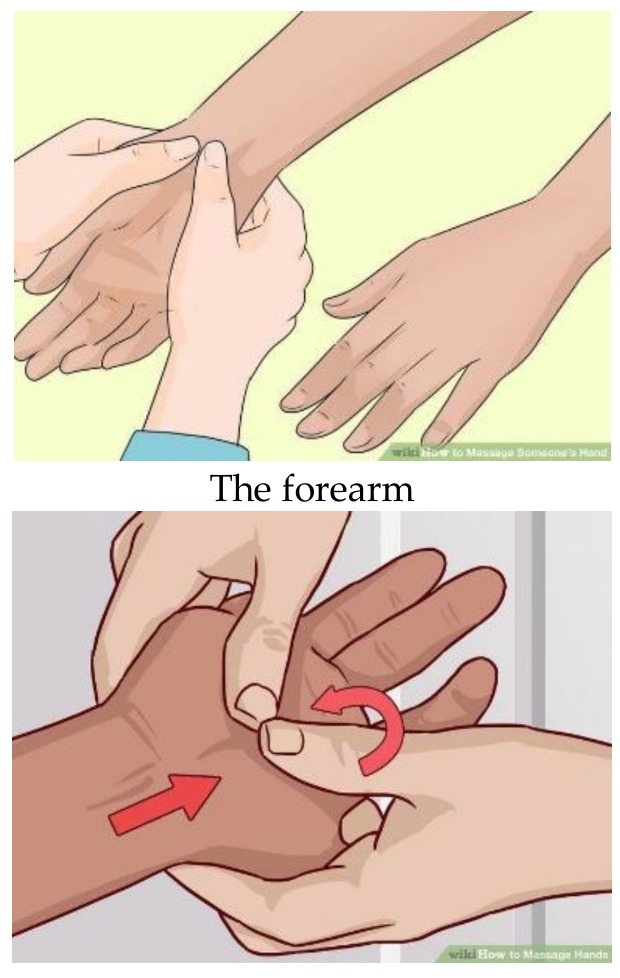

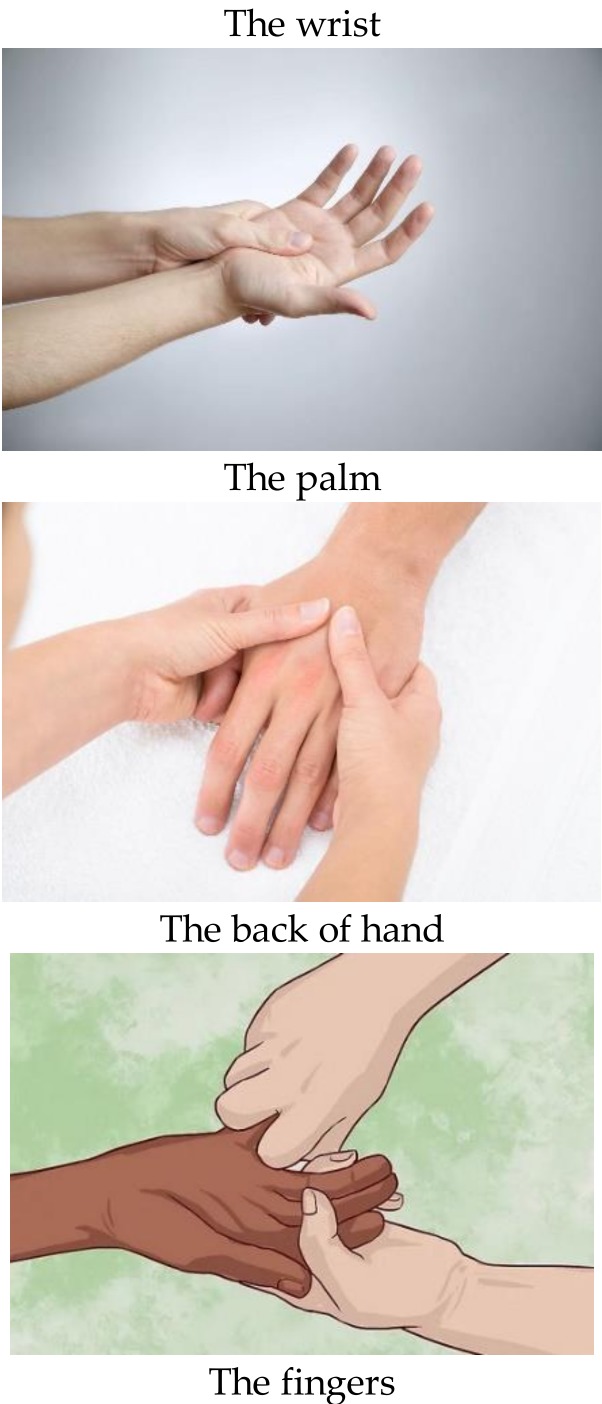
The points of hand reflexology.

Ingham’s method was used to apply massage [39] on the women’s hands for 10 min per hand. The massage was applied with the thumb, starting from the forearm, moving to the wrist, palm, back of the hand and, lastly, fingers. The patients received two minutes of reflexology massage at three reflexology points for the solar plexus, pituitary gland and heart with a moderate pressure on each hand. Firm downward pressure was applied with the thumb at the points for two minutes in every area. The pressure level was so that the upper thumb was colorless, but the patients did not feel discomfort. A circular rotational massage was performed at the same points (Figure 3).

### 2.11. Placebo Group

Conditions similar to the intervention groups were created, but a touch on thumbs without the stimulation of hand reflexology or acupressure was applied. 

### 2.12. Data Collection and Analysis

After patients’ allocation to the groups, anxiety was assessed before and after the interventions.

For data analysis, descriptive and inferential statistics were used via the Statistical Package for the Social Sciences software version 22 (SPSS Inc., Chicago, IL, USA). The Kolmogorov–Smirnov test examined the normal distribution of data, and Leven’s test helped assess homogeneity of the variance. A *p*-value less than 0.05 was considered statistically significant. 

### 2.13. Ethical Approval and Consent to Participate

This research was approved by the ethics committee affiliated with Shahed University (Decree code: IR.Shahed.REC.1395.171). Also, personal data for this research was collected with the individuals’ consent. 

## 3. Results

### 3.1. Demographic Characteristics

All 135 women with CAD were eligible for inclusion in this study and were assigned into one of the three groups. They agreed to participate and remained throughout the study process. Their mean age was 49.77 ± 6.67 years. The majority of them had the primary education level (36.3%), were married (84.4%), and housewives (88.1%) (*p* > 0.05). Except for the participants’ occupations, no statistically significant differences were found between the groups in terms of demographic characteristics including age, duration of stay in the CCU, education level, marital status, adequacy of income, residence place, smoking, history of hospital stay, and history of drug use (Table 1). 

### 3.2. Vital Signs

According to Table 2, no statistically significant differences in vital signs were reported between the groups after the interventions (*p* > 0.05). 

### 3.3. Anxiety Level

No statistically significant differences (*p* > 0.05) were reported between the groups before the interventions. Also, after the interventions, both immediately and half an hour later, the effects of both hand reflexology and acupressure on reduction of the participants’ anxiety were equal (*p* > 0.05). Comparison of anxiety levels before the interventions and at follow-ups within the groups and between the groups was presented in Table 3. 

## 4. Discussion

The effects of hand reflexology and acupressure on anxiety and vital signs in the women with CAD hospitalized in the CCU were examined in this study. The patients receiving either hand reflexology or acupressure demonstrated significant changes in their overall anxiety compared to the placebo group. In this study, the mean scores of anxiety after the interventions were reduced. The effect of both interventions on the reduction of anxiety was similar, indicating that acupressure and hand reflexology had moderate intensity effects. 

No similar studies on female patients with CAD were found in the literature to support the positive effect of hand reflexology on the reduction of anxiety. Available studies had a small sample size and patients with a different type of disease, various cultural–geographical characteristics and a different intervention place on the body, which might have led to controversies in results [36,40].

After the acupressure intervention, the mean score of anxiety in the patients was reduced, which was consistent with the results of other studies. For instance, Raja’i et al. (2015) reported that the use of acupressure reduced anxiety in patients undergoing coronary angiography [41]. However, it did not compare the effect of acupressure with other interventions on anxiety levels as carried out and reported in the present study. Valiee et al.’s study focused on the effect of acupressure on preoperative anxiety in patients undergoing abdominal surgery, indicating a significant reduction of anxiety compared with placebo [18].

In our study, the placebo group showed also significant changes in anxiety and vital signs, implying the Hawthorne phenomena. The presence of the data collector could have exacerbated the Hawthorne effect. It is estimated that the Hawthorne effect due to patients’ awareness of their situation can overestimate the effect of treatment by 17% [42,43]. The assessment of other factors influencing such changes in the placebo group needs further considerations by other researchers. 

Since this study was conducted in one hospital, and the interventions were carried out once and only on female patients, generalizability of findings to other settings should be done with caution. Longitudinal studies with a greater diversity of participants can further describe the full impact of the interventions with the consideration of possible gender differences affecting participants’ anxiety. More studies are also required to investigate the benefits of acupressure and hand reflexology for relieving psychological symptoms with the consideration of patients’ gender, age, and health issues. Research is also needed to examine the combination of interventions such as acupressure and hand reflexology on patients’ wellbeing and quality of life. 

## 5. Conclusions

According to this study, hand reflexology and acupressure had an equal effect on the reduction of anxiety in the women with CAD hospitalized in the CCU. No adverse events due to these interventions were reported, indicating that these nonpharmacologic methods were safe.

Acupressure and hand reflexology are simple to use, inexpensive and need no special instruments and devices. They can also become incorporated into the undergraduate and graduate nursing curricula. Nurses are encouraged to use these interventions to improve the quality of nursing care and reduce patients’ anxiety.

## Figures and Tables

**Table 1:** Comparison of demographic characteristics between the groups.

Demographic Data (Mean ± SD)	Hand Reflexology (*n* = 45)	Acupressure (*n* = 45)	Placebo (*n* = 45)	*p*-Value
**Age** (years)	50.16 ± 6.87	50.29 ± 6.47	48.87 ± 6.72	* F(2,132) = 0.61, *p* = 0.54
**Duration of stay in the CCU** (days)	2.57 ± 1.05	3.04 ± 1.08	2.73 ± 1.05	** H(2) = 5.01, DF = 2, *p* = 0.08
**Education level**	*n* (%)	*n* (%)	*n* (%)	
Illiterate	16 (35.6%)	10 (22.2%)	11 (24.5%)	*** X^2^(6) = 4.03, *p* = 0.67
Primary school	15 (33.3%)	16 (35.6%)	18 (40.0%)	
Pre-high school	9 (20.0%)	9 (20.0%)	10 (22.2%)	
High school	5 (11.1%)	10 (22.2%)	6 (13.3%)	
**Marital status**				
Married	38 (84.4%)	37 (82.2%)	39 (86.7%)	*** X^2^(2) = 0.33, *p* = 0.84
Widow	7 (15.6%)	8 (17.8%)	6 (13.3%)	
**Adequacy of income**				
Yes	9 (20.0%)	15 (33.3%)	15 (33.3%)	*** X^2^(4) = 4.75, *p* = 0.31
Somewhat	30 (66.7%)	21 (46.7%)	25 (55.6%)	
No	6 (13.3%)	9 (20.0%)	5 (11.1%)	
**Residence place**				
City	37 (82.2%)	34 (75.6%)	34 (75.6%)	*** X^2^(2) = 0.77, *p* = 0.68
Village	8 (17.8%)	11 (24.4%)	11 (24.4%)	
**Smoking**				
Yes	8 (17.8%)	6 (13.3%)	4 (8.9%)	*** X^2^(2) = 1.53, *p* = 0.46
No	37 (82.2%)	39 (86.7%)	41 (91.1%)	
**History of hospital stay**				
Yes	33 (73.3%)	32 (71.1%)	33 (73.3%)	*** X^2^(2) = 0.07, *p* = 0.96
No	12 (26.7%)	13 (28.9%)	12 (26.7%)	
**History of drug use**				
Yes	34 (75.6%)	31 (68.9%)	33 (73.3%)	*** X^2^(2) = 0.52, *p* = 0.77
No	11 (24.4%)	14 (31.1%)	12 (26.7%)	

* *p*-Value was calculated using the repeated-measures ANOVA test for between-group comparisons. Kolmogorov–Smirnov *p* > 0.5; ** *p*-value was calculated using the repeated measures Kruskal–Wallis test for between-group comparisons. Kolmogorov–Smirnov *p* < 0.5; *** *p*-value was calculated using the Chi-square test for between-group comparisons.

**Table 2:** Comparison of the mean scores of vital signs before the interventions and at two follow-ups within and between the groups.

Group (Mean ± SD)	Baseline	Immediately after the Intervention	Half an Hour after the Intervention	** *p*-Value
Hand reflexology (45) Heart rate	83.49 ± 10.77	83.51 ± 9.98	82.87 ± 11.04	Pillai’s Trace = 0.03, F(2,43) = 0.81, *p* = 0.44 Greenhouse. Geisser, F(2,88)= 0.50, *p* = 0.60
Acupressure (45) Heart rate	78.69 ± 13.80	77.16 ± 14.05	77.42 ± 13.36	Pillai’s Trace = 0.065, F(2,43) = 1.50, *p* = 0.23 Greenhouse. Geisser, F(2,88) = 2.30, *p* = 0.10
Placebo (45) Heart rate	83.42 ± 15.516	82.93 ± 14.35	82.51 ± 14.79	Pillai’s Trace = 0.02, F(2,43) = 0.06, *p* = 0.53 Greenhouse. Geisser, F(2,88) = 0.57, *p* = 0.56
* *p*-value	**F(2,132) = 1.86, 0.15**	**F(2,132) = 3.31, *p* =0.03**	**F(2,132) = 2.41, *p* =0.09**	Pillai’s Trace = 0.02, F(2,131) = 1.64, *p* = 0.19 Greenhouse. Geisser, F(2,264) = 2.25, *p* = 0.10
Hand reflexology (45) Respiratory rate	19.40 ± 1.94	18.89 ± 2.10	18.78 ± 1.46	Pillai’s Trace = 0.26, F(2,43) = 7.57, *p* = 0.002 Sphericity Assumed, F(2,88) = 7.50, *p* = 0.001
Acupressure (45) Respiratory rate	19.07 ± 1.42	18.91 ± 1.10	19.04 ± 1.18	Pillai’s Trace = 0.01, F(2,43) = 0.35, *p* = 0.70 Sphericity Assumed, F(2,88) = 0.7, *p* = 0.69
Control (45) Respiratory rate	19.40 ± 1.83	19.29 ± 2.09	19.22 ± 1.59	Pillai’s Trace = 0.02, F(2,43) = 0.58, *p* = 0.56 Sphericity Assumed, F(2,88) = 0.57, *p* = 0.56
* *p*-value	**F(2,132) = 0.54, *p* = 0.58**	**F(2,132) = 0.68, *p* = 0.50**	**F(2,132) = 1.11, *p* =0.33**	Pillai’s Trace = 0.24, F(2,131) = 1.64, *p* = 0.19 Greenhouse. Geisser, F(2,264) = 2.5, *p* = 0.12,
Hand reflexology (45) Mean arterial pressure	97.93 ± 19.09	100.02 ± 16.42	97.27 ± 17.45	Pillai’s Trace = 0.07, F(2,43) = 1.68, *p* = 0.19 Sphericity Assumed, F(2,88) = 1.46, *p* = 0.23
Acupressure (45) Mean arterial pressure	100.09 ± 18.12	102.89 ± 16.66	100.31 ± 16.39	Pillai’s Trace = 0.12, F(2,43) = 3.02, *p* = 0.06 Greenhouse. Geisser, F(2,88) = 2.46, *p* = 0.09
Control (45) Mean arterial pressure	97.47(19.91)	98.78(20.67)	97.82(21.31)	Pillai’s Trace = 0.02, F(2,43) = 0.61, *p* = 0.54 Greenhouse. Geisser, F(2,88) = 0.48, *p* = 0.59
* *p*-value	**F(2,132) = 0.24, *p* = 0.78**	**F(2,132) = 0.61, *p* = 0.54**	**F(2,132) = 0.34, *p* = 0.70**	Pillai’s Trace = 0.06, F(2,131) = 1.74, *p* = 0.10 Greenhouse. Geisser, F(2,264) = 3.89, *p* = 0.06

* *p*-Value was calculated using the one-way ANOVA test for between-group comparisons; ** *p*-value was calculated using the repeated-measure ANOVA test for within-group comparisons.

**Table 3:** Comparison of anxiety before the interventions and at two follow-ups within and between the groups.

Group (Mean ± SD)	Baseline	Immediately after the Intervention	Half an Hour after the Intervention	** *p*-Value
Hand reflexology (45) State-Anxiety	49.33 ± 11.30 Moderate to high anxiety	35.00 ± 9.03 Moderate to low anxiety	35.29 ± 8.96 Moderate to low anxiety	Pillai’s Trace = 0.86, F(2, 43) = 141.17, *p* = 0.001 Greenhouse. Geisser, F(2,88) = 258.49, *p* = 0.001
Acupressure (45) State-Anxiety	44.42 ± 9.22 Moderate to high anxiety	32.93 ± 11.66 Moderate to low anxiety	33.18 ± 11.81 Moderate to low anxiety	Pillai’s Trace = 0.60, F(2, 43) = 32.52, *p* = 0.001 Greenhouse. Geisser, F(2,88) = 61.13, *p* = 0.001
Control (45) State-Anxiety	48.22 ± 10.08 Moderate to high anxiety	43.91 ± 11.79 Moderate to high anxiety	43.84 ± 11.77 Moderate to high anxiety	Pillai’s Trace =0.21, F(2,43) =5.93, *p* = 0.005 Greenhouse. Geisser, F(2,88) = 11.64, *p* = 0.001
* *p*-value	**F(2,132) = 2.84, *p* = 0.06**	**F(2,132) = 12.87, *p* = 0.001** POWER = 0.99, η^2^ = 0.163, Cohen = 0.88, large. effect	**F(2,132) = 12.007, *p* = 0.001** POWER = 0.99, η^2^ = 0.154, Cohen = 0.85, large. effect	Pillai’s Trace =0.60, F(2,131) = 101.91, *p* = 0.001 Greenhouse. Geisser, F(2,264) = 198.10, *p* = 0.001, POWER = 1, η^2^ = 0.60, Cohen = 2.44, large. effect
Hand reflexology (45) Trait-Anxiety	48.36 ± 7.88 Moderate to high anxiety	37.89 ± 7.24 Moderate to low anxiety	37.93 ± 7.19 Moderate to low anxiety	Pillai’s Trace = 0.85, F(2,43) = 128.76, *p* = 0.001 Sphericity Assumed, F(2,88) = 262.11, *p* = 0.001
Acupressure (45) Trait-Anxiety	50.60 ± 9.38 Moderate to high anxiety	36.93 ± 11.82 Moderate to low anxiety	36.47 ± 11.70 Moderate to low anxiety	Pillai’s Trace = 0.67, F(2,43) = 44.28, *p* = 0.001 Sphericity Assumed, F(2,88)= 81.93, *p* = 0.001
Control (45) Trait-Anxiety	49.11 ± 9.34 Moderate to high-anxiety	45.49 ± 11.55 Moderate to high anxiety	45.58 ± 11.57 Moderate to high anxiety	Pillai’s Trace = 0.20, F(2,43) = 5.40, *p* = 0.008 Sphericity Assumed, F(2,88) = 9.24, *p* = 0.001
* *p*-value	**F(2,132) = 0.74, *p* = 0.47**	**F(2,132) = 9.11, *p* = 0.001** POWER = 0.97, η^2^ = 0.121, Cohen = 0.74, medium. effect	**F(2,132) = 10.01, *p* =0.001** POWER = 0.98, η^2^ = 0.132, Cohen = 0.77, medium. effect	Pillai’s Trace =0.59, F(2,131) = 97.68, *p* = 0.001 Greenhouse. Geisser, F(2,264) = 187.36, *p* = 0.001, POWER = 1, η^2^ = 0.58, Cohen = 2.35, large. effect
Hand reflexology (45)Total Anxiety	97.69 ± 17.37 Moderate to high anxiety	72.89 ± 15.17 Moderate to low anxiety	73.22 ± 15.13 Moderate to low anxiety	Pillai’s Trace = 0.90, F(2,43) = 195.99, *p* = 0.001 Sphericity Assumed, F(2,88) = 380.72, *p* = 0.001
Acupressure (45)Total Anxiety	95.02 ± 15.79 Moderate to high anxiety	69.87 ± 21.60 Moderate to low anxiety	69.64 ± 21.41 Moderate to low anxiety	Pillai’s Trace = 0.67, F(2,43) = 45.38, *p* = 0.001 Greenhouse. Geisser, F(2,88) = 84.69, *p* = 0.001
Control (45)Total Anxiety	97.33 ± 16.45 Moderate to high anxiety	89.40 ± 21.26 Moderate to high anxiety	89.42 ± 21.27 Moderate to high anxiety	Pillai’s Trace = 0.20, F(2,43) = 5.63, *p* = 0.007 Greenhouse. Geisser, F(2,88) = 11.15, *p* = 0.001
* *p*-value	**F(2,132) = 0.34, *p* = 0.70**	**F(2,132) =12.98, *p* = 0.001** POWER = 0.97, η^2^ = 0.164, Cohen = 0.88, large. effect	**F(2,132) = 13.14, *p* = 0.001** POWER = 0.99, η^2^ = 0.166, Cohen = 0.89, large. effect	Pillai’s Trace = 0.63, F(2,131) = 113.80, *p* = 0.001 Greenhouse. Geisser, F(2,264) = 226.53, *p* = 0.001, POWER = 1, η^2^ = 0.63, Cohen = 2.60, large. effect

* *p*-Value was calculated using the one-way ANOVA test for between-group comparisons; ** *p*-value was calculated using the repeated-measure ANOVA test for within-group comparisons; effect size ANOVA: $f=\sqrt{\frac{{ƞ}^{2}}{1-{ƞ}^{2}}}$, d = 2 * f.
